# Intravenous and oral whole body ketone dosimetry, biodistribution, metabolite correction and kinetics studied by (*R*)-[1-^11^C]β-hydroxybutyrate ([^11^C]OHB) PET in healthy humans

**DOI:** 10.1186/s41181-023-00198-z

**Published:** 2023-06-14

**Authors:** Thien Vinh Luong, Erik Nguyen Nielsen, Lise Falborg, Mette Louise Gram Kjærulff, Lars Poulsen Tolbod, Esben Søndergaard, Niels Møller, Ole Lajord Munk, Lars Christian Gormsen

**Affiliations:** 1 Department of Nuclear Medicine and PET Center, Aarhus University Hospital, Palle Juul-Jensens Boulevard 165, 8200 Aarhus N, Denmark; 2 Steno Diabetes Center Aarhus, Aarhus University Hospital, Palle Juul-Jensens Boulevard 11, 8200 Aarhus N, Denmark; 3 Medical/Steno Aarhus Research Laboratory, Department of Clinical Medicine, Aarhus University, Palle Juul-Jensens Boulevard 11, 8200 Aarhus N, Denmark; 4 Department of Endocrinology and Internal Medicine, Aarhus University Hospital, Palle Juul-Jensens Boulevard 161, 8200 Aarhus N, Denmark; 5 Department of Clinical Medicine, Aarhus University, Aarhus, Denmark

**Keywords:** Ketone metabolism, PET/CT, Biodistribution, Dosimetry, Kinetics

## Abstract

**Background:**

Ketones are increasingly recognized as an important and possibly oxygen sparing source of energy in vital organs such as the heart, the brain and the kidneys. Drug treatments, dietary regimens and oral ketone drinks designed to deliver ketones for organ and tissue energy production have therefore gained popularity. However, whether ingested ketones are taken up by various extra-cerebral tissues and to what extent is still largely unexplored. It was therefore the aim of this study to use positron emission tomography (PET) to explore the whole body dosimetry, biodistribution and kinetics of the ketone tracer (*R*)-[1-^11^C]β-hydroxybutyrate ([^11^C]OHB). Six healthy subjects (3 women and 3 men) underwent dynamic PET studies after both intravenous (90 min) and oral (120 min) administration of [^11^C]OHB. Dosimetry estimates of [^11^C]OHB was calculated using OLINDA/EXM software, biodistribution was assessed visually and [^11^C]OHB tissue kinetics were obtained using an arterial input function and tissue time-activity curves.

**Results:**

Radiation dosimetry yielded effective doses of 3.28 $\upmu$Sv/MBq (intravenous administration) and 12.51 $\upmu$Sv/MBq (oral administration). Intravenous administration of [^11^C]OHB resulted in avid radiotracer uptake in the heart, liver, and kidneys, whereas lesser uptake was observed in the salivary glands, pancreas, skeletal muscle and red marrow. Only minimal uptake was noted in the brain. Oral ingestion of the tracer resulted in rapid radiotracer appearance in the blood and radiotracer uptake in the heart, liver and kidneys. In general, [^11^C]OHB tissue kinetics after intravenous administration were best described by a reversible 2-tissue compartmental model.

**Conclusion:**

The PET radiotracer [^11^C]OHB shows promising potential in providing imaging data on ketone uptake in various physiologically relevant tissues. As a result, it may serve as a safe and non-invasive imaging tool for exploring ketone metabolism in organs and tissues of both patients and healthy individuals.

*Trial registration*

Clinical trials, NCT0523812, Registered February 10th 2022, https://clinicaltrials.gov/ct2/show/NCT05232812?cond=NCT05232812&draw=2&rank=1.

## Introduction

Ketones have recently been the focus of intensified research due to the possible beneficial cardiovascular and neurological effects associated with hyperketonemia (Ferrannini et al. 2016). Ketones are produced in the liver mainly from fatty acids during states of limited carbohydrate availability such as ketogenic dieting, fasting and prolonged exercise. Three different ketones are produced in humans in-vivo: 3-hydroxybutyrate (OHB), acetoacetate, and acetone, with OHB constituting the majority ($\sim \hspace{0.17em}$80%) of total circulating ketone concentration (Abdul Kadir et al. 2020). Ketones have been demonstrated to affect both metabolism and hemodynamics in a manner that could be advantageous in patients with a wide range of illnesses such as overweight, type 2 diabetes, cognitive impairment, and ischemic heart failure (Kosinski and Jornayvaz 2017; Mayer et al. 2014; Nielsen et al. 2019; Rusek et al. 2019). However, whether the putative beneficial effects of ketones are caused by a favorable ratio of oxygen expended to produce ATP (Veech et al. 2001), improved tissue perfusion (Svart et al. 2018) or whether ketones may simply add to the pool of available substrates for oxidation (Kadir et al. 2023) is presently largely unknown. Quantifying organ distribution, uptake and oxidation of ketones is therefore important to further our understanding of organ ketone utilization. However, measurement of ketone metabolism in the most relevant organs (heart and brain) is difficult and to some extent unethical in humans using conventional catheterization techniques, and relatively few (albeit high quality) studies of mostly cross-sectional nature therefore exist (Murashige et al. 2020). As an alternative to invasive studies, positron emission tomography/computed tomography (PET/CT) using the ketone radiotracers ([^11^C]OHB and [^11^C]acetoacetate) offers an attractive and non-invasive imaging alternative and consequently, some ketone PET studies have been performed to study the cerebral substrate metabolism in healthy subjects and in patients with neurodegenerative diseases (Fortier et al. 2019; Croteau et al. 2018; Blomqvist et al. 1995, 2002).

Despite the currently available PET studies of cerebral ketone metabolism, estimates of whole-body ketone radiotracer dosimetry, biodistribution and extracerebral kinetics in humans have not previously been published. It is therefore presently not documented if [^11^C]OHB and [^11^C]acetoacetate yield effective doses comparable to other ^11^C-labeled radiotracers (typically $\sim$ 5 μSv/MBq) (Zanotti-Fregonara et al. 2021).

Human biodistribution studies have only partly been explored with short duration PET scans (Cuenoud et al. 2020) and the relative avidity of various organs and tissues for ketones is therefore still inadequately characterized. In addition, only rodent PET studies of ketone body metabolism in the heart (Croteau et al. 2014) as well as in breast and prostate tumors (Authier et al. 2008) have to our knowledge been reported. Cardiac ketone metabolism is therefore mostly inferred from A–V balance studies (Murashige et al. 2020) or indirectly assessed using other radiotracers such as [^11^C]palmitate (Lauritsen et al. 2021) or [^18^F]FDG (Gormsen et al. 2017). It is therefore unknown whether cardiac ketone metabolism can be satisfactorily determined using simple 1- or 2-tissue compartmental modeling. Finally, although various ketone ester drinks are becoming increasingly popular both to enhance athletic performance (Evans et al. 2022) and to treat patients with e.g. heart failure (Selvaraj et al. 2020), no PET studies using oral ingestion of PET tracers have been performed.

The primary aim of this study was therefore to assess human [^11^C]OHB dosimetry and biodistribution after both intravenous and oral administration of the radiotracer. As a secondary aim, we wanted to calculate tissue and organ [^11^C]OHB kinetics using time-activity curves (TAC) obtained during the dosimetry studies and an input function measured from arterial sampling. Finally, we wanted to measure [^11^C]CO_2_ appearance in blood to supply robust estimates for metabolite correction.

## Materials and methods

### Study participants

The study included 6 healthy volunteers (3 men and 3 women) with a mean age of 56.3 years (range, 52–67 years) and a mean BMI of 27.7 kg/m^2^ (range, 24.6–30.9 kg/m^2^). All participants were screened for renal and cardiac diseases prior to inclusion and signed an informed consent for participation and publication, before being included in the study. The participant profile is provided in Table 1. The study site was The Department of Nuclear Medicine & PET Centre at Aarhus University Hospital.

**Table 1:** Participant profile

Age (years)	56.3 $\pm \hspace{0.17em}$5.4
BMI (kg/m^2^)	27.7 $\pm \hspace{0.17em}$2,6
Systolic blood pressure	132.8 $\pm \hspace{0.17em}$8.4
Diastolic blood pressure (mmHg)	85.7 $\pm \hspace{0.17em}$6.7
Cholesterol (mmol/L)	4.8 $\pm \hspace{0.17em}$0.3
HDL (mmol/L)	1.5 $\pm \hspace{0.17em}$0.3
LDL (mmol/L)	2.6 $\pm \hspace{0.17em}$0.4
Triglyceride (mmol/L)	1.5 $\pm \hspace{0.17em}$0.7
HbA1c (mmol/mol)	34.7 $\pm \hspace{0.17em}$6.1
Glucose (mmol/L)	5.9 $\pm \hspace{0.17em}$0.9
Alanine transaminase (U/L)	25.5 $\pm \hspace{0.17em}$10.0
Alkaline phosphatase (U/L)	68.31 $\pm \hspace{0.17em}$2.7
Hemoglobin (mmol/L)	8.3 $\pm \hspace{0.17em}$1.0

Values are mean $\pm$ SD

### Radiopharmaceutical preparation

**Fig. 1:**
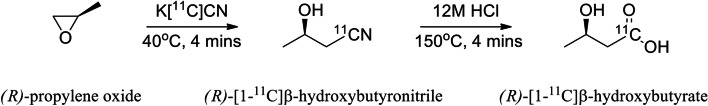
Production of (*R*)-[1-^11^C]$\beta$-hydroxybutyrate

The (*R*)-[1-^11^C]β-hydroxybutyrate was produced as previously reported with some modifications (Thorell et al. 1991). [^11^C]cyanide was produced from [^11^C]CO_2_ in a process cabinet (GE Healthcare) and delivered as NH_4_[^11^C]CN to an in-house built synthesis module. Initially, NH_4_[^11^C]CN (10–20 GBq) was trapped on a “cyanide trap” as previously reported (Drandarov et al. 2006), to facilitate the removal of ammonia, the presence of which would otherwise lead to hampering of the following hydrolysis step and formation of side products. Following elution of K[^11^C]CN from the “cyanide trap” to the reaction vessel with 400 µL sterile H_2_O, 400 µL of the precursor, (*R*)*-*propylene oxide, was added and the mixture was heated at 40 °C for 4 min in which time approximately 90% conversion to (*R*)-[1-^11^C]β-hydroxybutyronitrile was obtained. Next 2.4 mL 12 M hydrochloric acid was added to reach a final molarity of > 10 M of the hydrolysis mixture, which was subsequently heated at 150 °C for 4 min to achieve a conversion average of 50% (n = 19) from the intermediate (*R*)*-*[1-^11^C]β*-*hydroxybutyronitrile to the product (*R*)*-*[1-^11^C]β-hydroxybutyrate (Fig. 1). The crude acidic product mixture was purified by semi-preparative HPLC (PolymerX 10 × 250 mm, 10 µm, Phenomenex, Torrance) and the product fraction (~ 3 mL) was collected and passed through a 0.22 µm sterile filter and formulated in 7 mL sterile saline. (*R*)*-*[1-^11^C]β*-*hydroxybutyrate have been produced in radiochemical yields up to 2.8 GBq with decay corrected yields of up to 50%, radiochemical purities was > 97% and enantiomeric purities larger than 98%.

### whole-body PET imaging

#### Intravenous study

Dynamic whole-body (D-WB) PET with continuous-bed-motion was performed using a Biograph Vision 600 PET/CT system (Siemens) with 26.3 cm axial field of view. A low-dose CT (25 Ref mAs, 120 Ref. kV, CARE Dose4D, CARE kV, ADMIRE level 3) from vertex cranii to mid-thigh was acquired first. A mean bolus of 204.2 MBq (range, 182–222 MBq) of [^11^C]OHB was injected, and immediately afterwards 24 consecutive whole-body passes with increasing durations (to account for the short half-life of [^11^C]OHB) were obtained. The 24 passes had the following structure: 5 × 60 s, 5 × 120 s, 5 × 180 s, 6 × 300 s, and 3 × 600 s. The time intervals for the PET scans were therefore approximately 1–5, 6–15, 16–30, 31–60, and 61–90 min. The PET images were reconstructed using TrueX+TOF, 4 iterations and 5 subsets, 2-mm Gaussian post filter, and 440 × 400 matrix with voxel size 1.65^3^ mm^3^.

#### Oral study

An oral study was performed 120 min after the intravenous study using the same PET scanner. Participants rapidly ingested a mean oral dose of 96.11 MBq (range, 59.15–114.5 MBq) of [^11^C]OHB dissolved in water. A low-dose CT (as in the intravenous study) was performed, and 5 min after ingestion of the radiotracer, 23 consecutive whole-body continuous bed motion scans with increasing durations were obtained. The 23 scans had the following structure: 5 × 120 s, 5 × 180 s, 7 × 300, and 6 × 600 s. The time intervals were therefore approximately 6–15, 16–30, 31–65, and 66–125 min.

### Blood sampling and metabolite correction

During the D-WB PET scans, manual blood samples were taken to measure arterial radioactivity for the input function. Both plasma and whole-blood radioactivity were measured in a well counter (Hidex AMG). We collected 40 manual blood samples during the intravenous studies and 23 manual blood samples during the oral studies. In addition, 8 manual venous blood samples were taken to correct for conversion of [^11^C]OHB to [^11^C]CO_2_, the only quantitatively important metabolite that has to be taken into account during ketone PET studies lasting less than 120 min (Cotter et al. 2013).

To determine [^11^C]CO_2_ in blood, two 0.5 mL venous whole blood samples were drawn simultaneously during the intravenous study (5, 10, 15, 20, 30, 50, 70, and 90 min after administration of [^11^C]-OHB). They were then placed in tubes containing 1.5 mL of isopropyl alcohol and 0.5 mL of 0.9 M sodium bicarbonate. Sample 1 was treated with 0.5 mL of 0.1 N sodium hydroxide and sample 2 was mixed with 0.5 mL of 6 N hydrochloric acid and vigorously stirred with a magnet for 10 min at room temperature. The radioactivity of each sample was then measured in a well counter for 1 min, and the data was corrected for decay to a common time point. Radioactivity in sample 2 divided by sample 1 was then considered the parent fraction. A correction curve was established by fitting the parent fraction curve using a monoexponential function and applied to the blood input function.

### Defining organ and tissue volumes of interest on the PET images

All D-WB PET images were visually inspected in PMOD 4.3 (Zurich, Switzerland). For PET scans following i.v. injection, the source organs for the dosimetry calculations were the brain, salivary glands, heart, liver, spleen, kidneys, pancreas, red marrow, and urinary bladder contents. For PET scans following oral ingestions, the source organs for the dosimetry calculations were the stomach contents, small intestine, heart, liver, kidneys, and salivary glands.

Volumes of interest (VOIs) were defined in PMOD 4.3. A sphere or box was drawn as large as possible for each organ to encompass the entire organ. The VOIs were drawn from summed images from 20 min until the end of scan of the intravenous and oral administration, respectively. This was done to minimize the spill-in from the blood pool and background. In difficult cases, the CT scan was used to aid the correct anatomical placement of the sphere/box. Thresholding was set as low as possible and was subsequently used to segment the organs within the bounding sphere/box. For the heart (left ventricle), pancreas (cauda pancreatis), red marrow (one lumbar vertebra), and skeletal muscle (musculus vastus lateralis) a smaller VOI was drawn within the organ. The VOI representing the brain was drawn by utilizing the full brain atlas supplied by PMOD.

### Biodistribution and dosimetry

For each source organ, the time course of the non-decay-corrected total radioactivity was normalized to the administered activity and recalculated to time courses of percentage injected activity. Time-integrated activity coefficients (TIACs) were computed using the trapezoidal integration method to calculate the area under the curves, assuming only physical decay after the last scan without further biological clearance. For oral administrations, we assumed that 100% of the tracer was in the stomach contents at the scan start. The remaining TIAC was determined by subtracting the TIACs of individual source organs from the overall TIAC of the entire body (excluding voiding), which for ^11^C is 0.49 h. The TIACs of the source organs and the remaining TIAC were utilized in OLINDA/EXM 2.0 (HERMES Medical Solution AB, Sweden (Stabin and Siegel 2018) to calculate the absorbed doses (μGy/MBq) for each organ and the effective dose (μSv/MBq) using anthropomorphic human body phantoms. These phantoms were designed based on organ masses from ICRP89 (Valentin 2002) and tissue weighting factors from ICRP103 (Valentin 2007). The organ doses and effective dose results are provided for the reference gender-averaged adult in accordance with ICRP103 guidelines.

### Kinetic analyses

All kinetic analyses on data from the intravenous study were done using PMOD 4.3 software. Kinetic analysis on data obtained from the oral study was excluded from kinetic analysis since most of the tracer was retained in the ventricle and small intestine. Arterial plasma input functions were corrected for metabolites. Organ and tissue radiotracer kinetics were analyzed using reversible or irreversible models and, in all cases, the Akaike Information Criterion (AIC) favored a reversible model. For all organs and tissues, we compared a 1-tissue with a 2-tissue compartment model using fixed estimates of tissue blood volume fraction (vB). The 2-tissue compartment model yielded the best AIC for all organs and tissues (except for the brain) and we therefore proceeded to perform full kinetic analysis using this model assuming reversible kinetics. Since we were limited by the relatively small axial field-of-view of the scanner, and utilized continuous-bed-motion to cover the whole body, we were precluded from obtaining sufficiently detailed radiotracer first pass data in all organs and tissues. Consequently, we opted to use a fixed tissue vB according to published data for the heart (Colbert et al. 2021), brain (Muzi et al. 2009), kidneys (O'Connor 2006), liver (Taniguchi et al. 1996), pancreas (Delrue et al. 2012), red marrow (Marenzana and Arnett 2013), and skeletal muscle (Saltin et al. 1998). Since no data exists on the vB in salivary glands, it was estimated to be 5%. In addition to compartment modelling, we estimated brain radiotracer uptake using the initial 10 min and assuming irreversible uptake to be able to compare our results with those previously obtained by other groups using [^11^C]-acetoacetate in particular (Courchesne-Loyer et al. 2017; Castellano et al. 2015).

## Results

The mean and standard deviation of the administered mass of [^11^C]OHB was 5.59$\pm$3.27 μg (range, 1.00–9.24 μg) during the intravenous study and 35.74$\pm$62.97 μg (range, 2.38–162.0 μg) during the oral study. The mean and standard deviation of the administered activity was 204.2 $\pm$ 16.15 MBq (range, 182–222 MBq) during the intravenous study and 96.11$\pm$21.32 MBq (range, 59.15–114.5 MBq) during the oral study. There were no adverse or clinically detectable pharmacologic effects in any of the 6 subjects. No significant changes in vital signs or the results of laboratory studies or electrocardiograms were observed.

### Biodistribution and dosimetry

#### Intravenous study

**Fig. 2:**
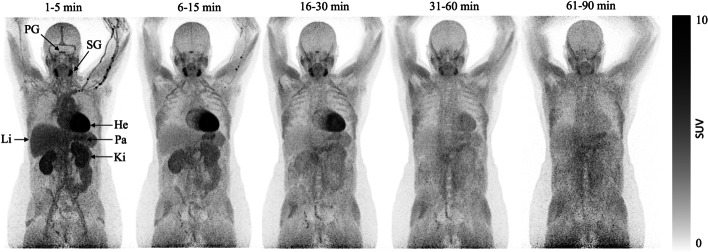
Summed PET scans of a representative patient after intravenous injection of 182 MBq of [^11^C]OHB. Abbreviations: He = heart; Ki = kidney; Li = liver; Pa = pancreas; PG = pituitary gland; SG = salivary gland

**Fig. 3:**
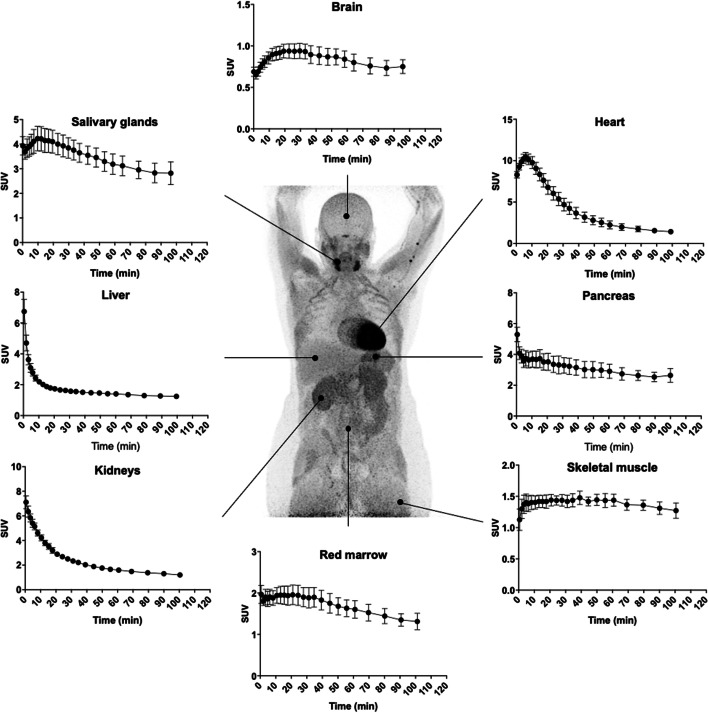
SUV curves of target organs after intravenous injection with [^11^C]OHB. Error bars are $\pm$ SEM

Representative summed whole body PET scans at different time points are presented in Fig. 2. As seen, radiotracer rapidly accumulated in the heart, kidneys and submandibular glands and also in the pituitary. Blood activity was only visible for the first 5 min. Over the course of the following 90 min, radiotracer activity visibly decreased in these organs and appeared to be redistributed towards skeletal muscle. Little activity was noted in the brain. Tissue and organ TACs are presented in Fig. 3. Heart, brain, salivary glands and to a lesser extent skeletal muscle and red marrow TAC had well defined peaks with the greatest activity noted in the heart followed by the salivary glands. Liver, kidney and pancreas TAC did not have well defined peaks, since radioactivity decreased throughout the study.

**Fig. 4:**
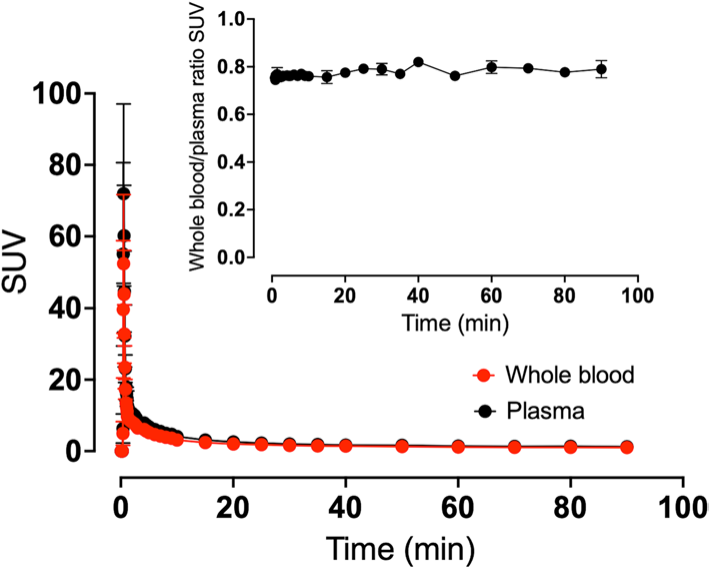
SUV of whole-blood and plasma activity after intravenous injection of the tracer. The inserted graph shows wholeblood/plasma ratio. Error bars are $\pm$ SEM

Rapid clearance of radiotracer from the circulation was evident as depicted in Fig. 4. The whole-blood to plasma activity ratio was close to 0.8 from the start of the study and remained constant throughout the scan. Radiation dosimetry of [^11^C]OHB after an intravenous injection is presented in Table 2. In line with tissue TAC, absorbed doses were found to be greatest in the heart, pancreas, kidneys, spleen, and salivary glands, while doses to the liver, red marrow, and urinary bladder also were notable yielding an effective dose of 3.28 $\upmu$Sv/MBq.

**Table 2:** Radiation dosimetry of [^11^C]OHB. Absorbed dose estimates and effective doses in humans

Target organ	Intravenous ($\upmu$Sv/MBq)	Oral ($\upmu$Sv/MBq)
Adrenals	4.03 $\pm \hspace{0.17em}$0.24	5.41 $\pm \hspace{0.17em}$1.13
Brain	2.21 $\pm \hspace{0.17em}$0.17	1.82 $\pm \hspace{0.17em}$0.41
Breasts	2.94 $\pm \hspace{0.17em}$0.03	2.32 $\pm \hspace{0.17em}$0.22
Esophagus	3.21 $\pm \hspace{0.17em}$0.04	3.95 $\pm \hspace{0.17em}$0.69
Eyes	2.73 $\pm \hspace{0.17em}$0.02	1.82 $\pm \hspace{0.17em}$0.40
Gallbladder wall	3.54 $\pm \hspace{0.17em}$0.08	3.71 $\pm \hspace{0.17em}$0.22
Left colon	3.68 $\pm \hspace{0.17em}$0.03	5.40 $\pm \hspace{0.17em}$0.57
Small intestine	3.50 $\pm \hspace{0.17em}$0.03	28.47 $\pm \hspace{0.17em}$9.54
Stomach wall	3.63 $\pm \hspace{0.17em}$0.07	77.70 $\pm \hspace{0.17em}$47.19
Right colon	3.54 $\pm \hspace{0.17em}$0.02	3.32 $\pm \hspace{0.17em}$0.53
Rectum	3.49 $\pm \hspace{0.17em}$0.05	2.86 $\pm \hspace{0.17em}$0.54
Heart wall	8.11 $\pm \hspace{0.17em}$1.66	6.38 $\pm \hspace{0.17em}$1.36
Kidneys	6.45 $\pm \hspace{0.17em}$0.99	4.68 $\pm \hspace{0.17em}$0.60
Liver	3.29 $\pm \hspace{0.17em}$0.68	3.69 $\pm \hspace{0.17em}$0.68
Lungs	3.18 $\pm \hspace{0.17em}$0.02	3.21 $\pm \hspace{0.17em}$0.26
Ovaries	3.89 $\pm \hspace{0.17em}$0.05	3.40 $\pm \hspace{0.17em}$0.61
Pancreas	8.22 $\pm \hspace{0.17em}$2.14	9.15 $\pm \hspace{0.17em}$2.95
Prostate	3.09 $\pm \hspace{0.17em}$0.04	2.42 $\pm \hspace{0.17em}$0.44
Salivary glands	5.91 $\pm \hspace{0.17em}$2.26	2.25 $\pm \hspace{0.17em}$0.92
Red marrow	4.23 $\pm \hspace{0.17em}$0.43	2.42 $\pm \hspace{0.17em}$0.17
Osteogenic cells	3.10 $\pm \hspace{0.17em}$0.24	1.87 $\pm \hspace{0.17em}$0.18
Spleen	7.75 $\pm \hspace{0.17em}$3.06	6.39 $\pm \hspace{0.17em}$1.99
Testes	2.67 $\pm \hspace{0.17em}$0.04	1.78 $\pm \hspace{0.17em}$0.40
Thymus	3.35 $\pm \hspace{0.17em}$0.05	2.62 $\pm \hspace{0.17em}$0.21
Thyroid	3.04 $\pm \hspace{0.17em}$0.03	2.16 $\pm \hspace{0.17em}$0.34
Urinary bladder wall	3.47 $\pm \hspace{0.17em}$0.53	2.52 $\pm \hspace{0.17em}$0.56
Uterus	3.83 $\pm \hspace{0.17em}$0.05	3.59 $\pm \hspace{0.17em}$0.68
Total body	3.18 $\pm \hspace{0.17em}$0.00	3.13 $\pm \hspace{0.17em}$0.03
Effective dose	3.28 $\pm \hspace{0.17em}$0.09	12.51 $\pm \hspace{0.17em}$5.90

Mean intravenous dose was 204.2 MBq (range, 182–222) and mean oral dose was 96.11 MBq (range, 59.15–114.5). Values are mean $\pm$ SD

#### Oral study

**Fig. 5:**
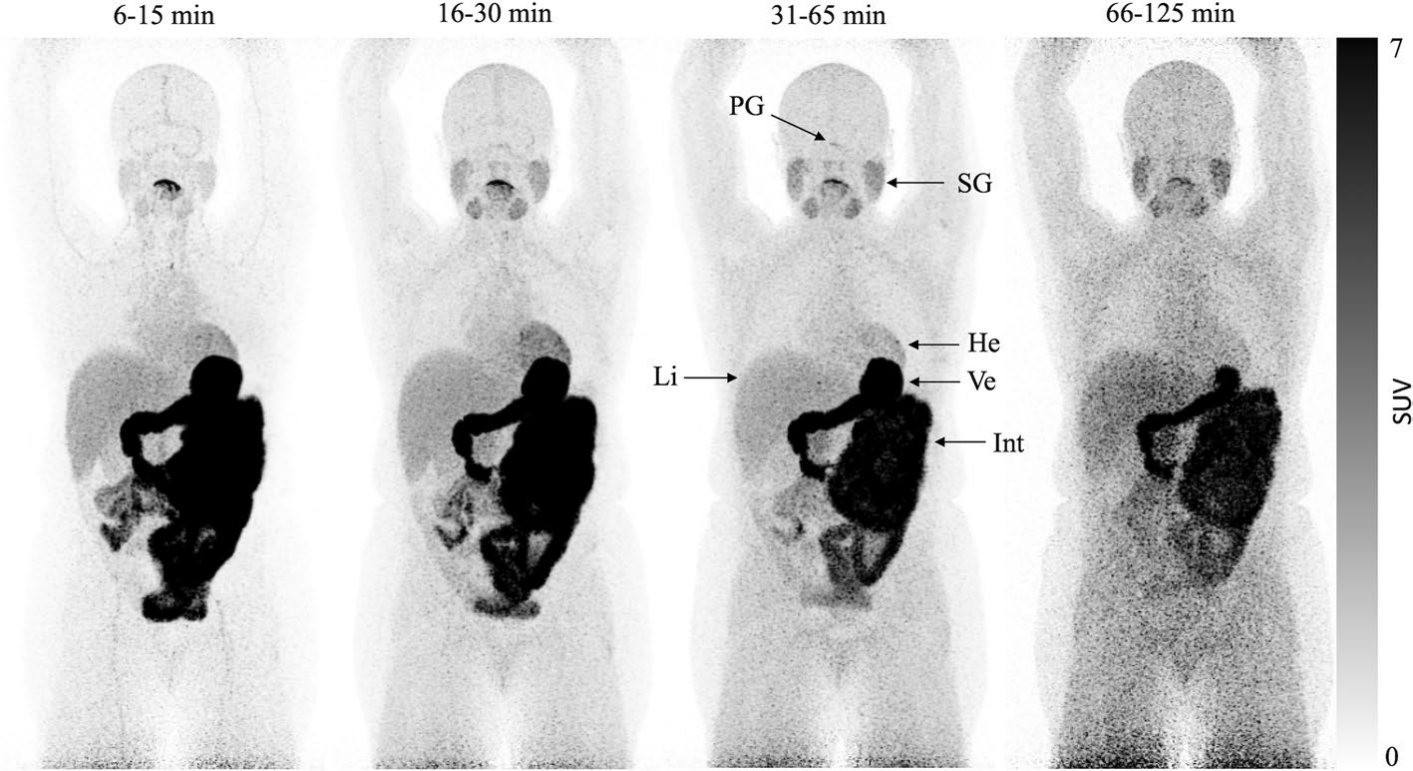
Summed PET scans of a representative patient after oral ingestion of 114 MBq [^11^C]OHB. Abbreviations: He = heart; Int = small intestine; Li = liver; PG = pituitary gland; SG = salivary gland; Ve = ventricle

**Fig. 6:**
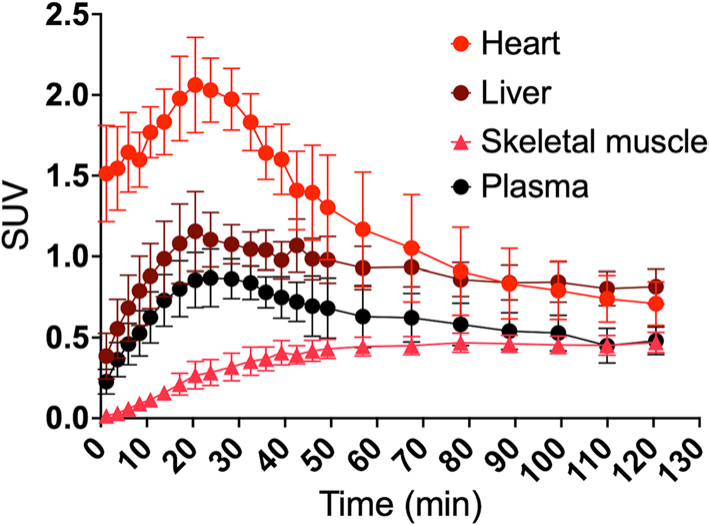
SUV curves after oral ingestion of [^11^C]OHB. Error bars are $\pm$ SEM

Representative summed images after an oral ingestion of the radiotracer are shown in Fig. 5. Radiotracer activity was visible in all relevant target organs within the first 15 min after ingestion showing a similar pattern to that following intravenous administration. Thus, heart activity was visibly decreasing after an initial peak at ~ 30 min and skeletal muscle activity appeared to increase throughout the study. Contrasting the intravenous administration, peaks of liver and kidney activity could be detected after 20 min (Fig. 6) followed by a gradual decline in activity. Not surprisingly, most of the radiotracer was retained in the GI-tract throughout the scan. Oral ingestion of radiotracer yielded an effective dose of 12.51 $\upmu$Sv/MBq with the stomach, intestines and pancreas identified as the most critical organs.

### Metabolite analysis

**Fig. 7:**
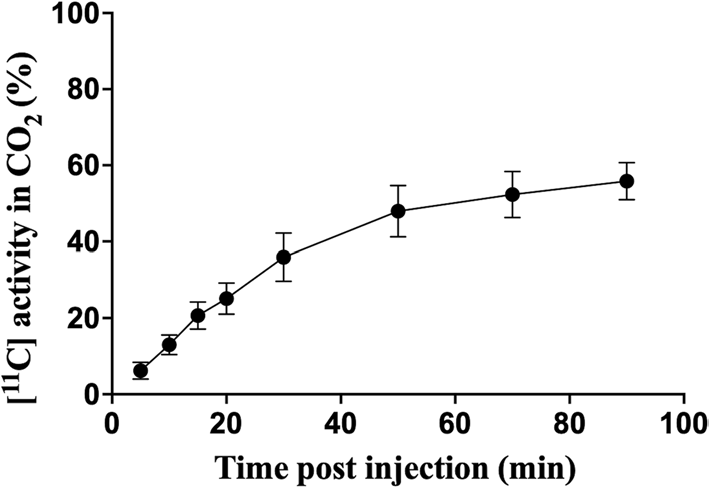
Whole-blood activity of [^11^C] as CO_2_ measured at 5 min (6.16 $\pm \hspace{0.17em}$5.46%), 10 min (12.99 $\pm \hspace{0.17em}$6.33%), 15 min (20.67 $\pm \hspace{0.17em}$8.73%), 20 min (25.10 $\pm \hspace{0.17em}$10.03%), 30 min (35.91 $\pm \hspace{0.17em}$15.48%), 50 min (47.97 $\pm \hspace{0.17em}$16.56%), 70 min (52.32 $\pm \hspace{0.17em}$14.78%) and 90 min (55.85 $\pm \hspace{0.17em}$11.90%). Values are mean $\pm$ SEM

The fraction of [^11^C]CO_2_ in circulating plasma [^11^C] activity after an intravenous injection of [^11^C]OHB is presented in Fig. 7. The [^11^C]CO_2_ fraction increased throughout the study measured at following time points 5 min (6.16 $\pm \hspace{0.17em}$5.46%), 10 min (12.99 $\pm \hspace{0.17em}$6.33%), 15 min (20.67 $\pm \hspace{0.17em}$8.73%), 20 min (25.10 $\pm \hspace{0.17em}$10.03%), 30 min (35.91 $\pm \hspace{0.17em}$15.48%), 50 min (47.97 $\pm \hspace{0.17em}$16.56%), 70 min (52.32 $\pm \hspace{0.17em}$14.78%), and 90 min (55.85 $\pm \hspace{0.17em}$11.90%) indicating a plateau around 55%.

### Kinetic analyses

All calculated transfer rate constants obtained using compartmental modeling during intravenous administration of [^11^C]OHB are presented in Table 3. K1 values (representing tissue flow and extraction) were greatest in the heart, followed by the salivary glands and pancreas. By contrast, the K1 value for the brain was markedly lower using the full data set and a reversible 1-tissue model. In fact, skeletal muscle K1 values were 4 times higher than those of the brain. Using the initial 10 min of the scan and assuming irreversible radiotracer kinetics (as done in previous brain ketone PET studies), we calculated the brain Patlak K-value to 0.0098 mL/mL/min. The k3 values (representing the rate of binding, transport or trapping of [^11^C]OHB) were similar across all analyzed organs.

**Table 3:** [^11^C]OHB kinetics (intravenous administration)

	K1 (mL/mL/min)	k2 (1/min)	k3 (1/min)	k4 (1/min)	Ki (mL/100 g/min)	Vt (mL/mL)
Heart wall 2 T	0.49 $\pm \hspace{0.17em}$0.20	0.42 $\pm \hspace{0.17em}$0.35	0.18 $\pm \hspace{0.17em}$0.14	0.16 $\pm \hspace{0.17em}$0.03	13.85 $\pm \hspace{0.17em}$2.69	3.03 $\pm \hspace{0.17em}$0.47
Skeletal muscle 2T	0.04 $\pm \hspace{0.17em}$0.03	0.19 $\pm \hspace{0.17em}$0.15	0.17 $\pm \hspace{0.17em}$0.13	0.02 $\pm \hspace{0.17em}$0.02	1.54 $\pm \hspace{0.17em}$0.57	2.50 $\pm \hspace{0.17em}$1.42
Salivary glands 2T	0.26 $\pm \hspace{0.17em}$0.08	0.75 $\pm \hspace{0.17em}$0.31	0.19 $\pm \hspace{0.17em}$0.04	0.02 $\pm \hspace{0.17em}$0.01	5.41 $\pm \hspace{0.17em}$1.93	3.94 $\pm \hspace{0.17em}$1.77
Red marrow 2T	0.12 $\pm \hspace{0.17em}$0.08	0.67 $\pm \hspace{0.17em}$0.37	0.20 $\pm \hspace{0.17em}$0.09	0.02 $\pm \hspace{0.17em}$0.004	2.43 $\pm \hspace{0.17em}$0.84	1.86 $\pm \hspace{0.17em}$0.67
Pancreas 2T	0.20 $\pm \hspace{0.17em}$0.08	0.58 $\pm \hspace{0.17em}$0.36	0.15 $\pm \hspace{0.17em}$0.10	0.02 $\pm \hspace{0.17em}$0.00	4.52 $\pm \hspace{0.17em}$2.30	4.42 $\pm 2.$ 77
Brain 1T	0.01 $\pm \hspace{0.17em}$0.001	0.01 $\pm \hspace{0.17em}$0.01				4.65 $\pm \hspace{0.17em}$7.25
Brain (10min)					0.98 $\pm \hspace{0.17em}$0.12	

The Patlak analysis of brain kinetics only included the first 10 min of the scan. Values are presented as mean $\pm$ SD

1 T = 1-tissue-compartment model; 2 T = 2-tissue-compartment model; Vt = total distribution volume. Ki = K1*k3/(k2 + k3)

## Discussion

Although ketone PET tracers have previously been used in brain research, no comprehensive dosimetry, whole body biodistribution or extra-cerebral tissue and organ tracer kinetics in humans have to our knowledge been published. These issues are all addressed in this paper.

### ***[***^***11***^***C]OHB dosimetry favors the use of the radiotracer in healthy individuals***

As clearly seen after intravenous administration of [^11^C]OHB, the radiotracer was distributed to most tissues in the body and radiation dosimetry therefore turned out to be a modest 3.3 μSv/MBq. We used ~ 200 MBq for each injection, which yielded acceptable counts for the input function, tissue radioactivity in target organs, and metabolite correction. Using 200 MBq of [^11^C]OHB for i.v. studies therefore results in a total radiation dose from the PET scan well below 1 mSv (0.66 mSv). Thus, a limited field-of-view [^11^C]OHB PET/CT is associated with such a low amount of ionizing radiation that it can safely be used for biomedical research, even in healthy, younger volunteers and also in serial PET scans (Radiological Protection in Biomedical Research. A report of Committee 3 adopted by the International Commission on Radiological Protection xxxx). This is important for several reasons. First, ketone metabolism in healthy individuals is currently a hot topic with both nutritional scientists and lay people arguing that at least some degree of ketosis may be beneficial for the general health of most people (Ferrannini et al. 2016; Zinman et al. 2015). The acceptable dosimetry of [^11^C]OHB allows for cross-sectional ketone metabolism studies of different metabolic phenotypes and across different ages and may therefore shed light on possible differences in organ and tissue ketone uptake capacity. For example, it is possible that cardiac ketone uptake decreases with age depriving the heart of an efficient source of energy. It is also possible that a sedentary lifestyle alters the capacity to utilize ketones in skeletal muscle. Second, serial [^11^C]OHB PET scans can be performed in patients undergoing various treatments known to affect circulating ketone levels but where it is currently unknown to what extent the treatment affects organ ketone metabolism. In this context, it has been of interest to our group whether SGLT2 inhibitors affect cardiac ketone metabolism and we have indirectly shown that this is likely the case (Lauritsen et al. 2021). Third, multitracer PET studies involving [^11^C]OHB will be feasible and acceptable e.g. in patients with cardiac diseases enabling a comprehensive overview of myocardial substrate metabolism, perfusion and function.

The effective dose after oral administration was less favorable but was still sufficiently low to allow using the tracer in patient studies. This may be particularly interesting in patients with heart failure, where it is currently debated whether oral ingestion of ketone esters may enhance cardiac output and function (Selvaraj et al. 2020). Even a modest dose of ~ 100 MBq [^11^C]OHB ingested orally resulted in acceptable target-to-background radioactivity ratios in the heart and simple SUV measurements performed at peak cardiac radioactivity (20 min post ingestion) may likely serve as a surrogate marker of cardiac ketone metabolism of recently ingested ketones.

### ***[***^***11***^***C]OHB biodistribution***

Overall, [^11^C]OHB was distributed to most tissues in the body in a manner consistent with the ubiquitous utilization of ketones for energy production. Thus, the tracer was clearly taken up by omnivorous energy consuming tissues such as the heart, salivary glands and kidneys, and was also slowly accumulating in skeletal muscle which is known to have the capacity to oxidize ketones—especially during exercise but also at rest (Evans et al. 2017). However, much to our surprise, [^11^C]OHB uptake in the brain was markedly less than expected for an organ that has been demonstrated to avidly consume ketones – at least under conditions of prolonged ketosis (Cahill 2006). It is possible that the lack of brain [^11^C]OHB uptake observed in this study was related to the participating subjects. In contrast to earlier brain ketone tracer studies, our participants had not undergone a lengthy ketogenic diet or long-term sipping of oral ketone esters before the study. Although they were overnight fasted, the length of the fast (10 h) was also unlikely to induce any increase in circulating ketones. Our participants therefore most likely had ketone levels below 0.1 mM, which is a major difference to the general levels of patients previously investigated (from 1 to 5 mM) (Cahill 2006; Puchalska and Crawford 2017). It is therefore plausible that the brain uptake [^11^C]OHB had been different if we had investigated patients in during prolonged ketosis. Of note, the pituitary gland was clearly taking up [^11^C]OHB to a much higher degree than the brain, and since the pituitary is located outside of the blood–brain barrier, it is possible that the rate limiting factor is located in the blood–brain barrier.

The biodistribution of [^11^C]OHB also revealed that although the liver lacks the enzymatic apparatus to oxidize ketones (Abdul Kadir et al. 2020), it does take up ketones from the circulation. Hepatic ketone uptake is facilitated by ketone transporting mono carboxyl transporters (MCTs) of which there are several on the hepatocytes (Felmlee et al. 2020). Since ketones are also produced in the liver, it is unclear what purpose reuptake from the circulation serves, but it could be as a negative feedback mechanism or simply exist in an equilibrium with the circulation. Kidney and bladder [^11^C]OHB uptake was as expected during conditions of low ketones. Thus, avid [^11^C]OHB activity was noted in the kidneys shortly after administration of the tracer reflecting the ability of the kidneys to both filtrate and oxidize ketones (Palmer and Clegg 2021). No activity was observed in the bladder consistent with complete reabsorption of the tracer following filtration facilitated by tubular MCTs (Cuenoud et al. 2020).

### ***[***^***11***^***C]OHB plasma/whole blood activity, metabolite correction and tissue kinetics***

Arterial whole blood and plasma were sampled for radioactivity measurements to determine whether [^11^C]OHB is taken up by red blood cells (RBCs). The ratio was stable at ~ 0.8 and thus well above the 0.55 (assuming a hematocrit of 45%) observed for radiotracers that are not taken up by RBCs. At first, this may appear counterintuitive since RBC do not contain mitochondria and therefore cannot oxidize ketones. However, membranes on human RBC contain MCT1 transporters for secretion of the end product of red blood cell glycolysis, lactate (Szabó et al. 2021). MCT1 transporters are bidirectional (Felmlee et al. 2020) and the flux is passive. Since MCT1 also transports ketones, it is plausible that RBC uptake of ketones (and therefore also of [^11^C]-OHB) simply reflects a concentration equilibrium. Regardless of the uptake mechanism, the whole blood/plasma activity ratio was 0.78 $\pm \hspace{0.17em}$0.02 from 5 to 90 min post injection and did not differ much between participants. Previously, the WB/plasma ratio has been reported as 0.82 for 10 min after injection of [^11^C]OHB and our data thus falls well in line with this (Blomqvist et al. 1995). This presents an opportunity to use image derived measurements of arterial activity for the input function (IDIFs), and can thus spare participants in research studies an arterial catheter.

Following tissue uptake, [^11^C]OHB is rapidly oxidized and the most important metabolite, [^11^C]CO_2_ is subsequently secreted into the blood stream. Consistent with this, we observed an increasing fraction of [^11^C]CO_2_ in plasma from 5 min (6.16 $\pm \hspace{0.17em}$5.46%) to 90 min (55.85 $\pm \hspace{0.17em}$11.90%) (Fig. 7). To our knowledge, [^11^C]OHB metabolite data have not previously been published, although others have estimated the fraction of [^11^C]CO_2_ to be ~ 6% of plasma radioactivity for the first 10 min after injection the ketone radiotracer [^11^C]acetoacetate (Castellano et al. 2015). Our data therefore indicates that the contribution of metabolites to plasma activity is greater than previously thought, and it is our belief that plasma [^11^C]CO_2_ fraction will have to be taken into account in order to correctly estimate the input function, particularly in studies lasting more than 10 min.

Analysis of [^11^C]OHB tissue kinetics were based on fewer data points than usually obtained in dynamic studies, since we used a series of WB passes on a PET scanner with a 26.3 cm axial field-of-view. Tissue TAC were therefore acquired from continuous bed motion passes with increasing durations and with significantly higher temporal resolution for the first minute post injection. We were therefore unable to obtain meaningful estimates of tissue Vb and instead used previously published Vb estimates. However, tissue TAC were generally well defined with prominent peaks and subsequent decays resulting in consistent estimates of transfer rate constants. Of interest, [^11^C]OHB kinetics were best described by reversible models with a 2-tissue compartmental model fitting the data well in most tissues and organs. Rate constants were within the range of the expected for the few organs that have previously been studied by ketone tracers. For example, K1 values in the heart averaged 0.49 mL/mL/min reflecting the relatively significant myocardial perfusion at rest and an extraction of [^11^C]OHB tracer approaching 50%. This estimate is consistent with previous measures of cardiac ketone extraction obtained by coronary sinus sampling in humans and also corroborates the notion of the heart as a major target organ for ketone body consumption (Murashige et al. 2020). Myocardial [^11^C]OHB TAC rapidly decreased after the initial peak resulting in quite high k2 values presumably reflecting mitochondrial oxidation, although this has not yet been properly explored as it has for [^11^C]acetate (Harms et al. 2018). The myocardial k3 and k4 rate constants may either represent shuttling of the [^11^C] isotope from the TCA cycle into other metabolic pathways or possibly binding to extra- or intracellular receptors. In the brain, our K1 and Patlak Ki values were also in line with those previously reported using [^11^C]OHB (Blomqvist et al. 1995) in healthy individuals (0.011 mL/mL/min) but were only about 33% of what has been reported using another ketone radiotracer, [^11^C-acetoacetate] (Courchesne-Loyer et al. 2017; Castellano et al. 2015). It is possible, that this discrepancy can be ascribed to the short acquisition protocol used in the [^11^C]acetoacetate studies, differences in metabolite correction or the participant demographics. In addition, it should be noted that brain radioactivity was rather low in comparison with other ketone oxidizing organs such as the heart or the kidneys. Consequently, [11C]CO_2_ excreted from other tissues could have contributed in a non-negligible manner to the brain radioactivity concentration. In fact, to accurately describe brain OHB kinetics, a parallel kinetic model encompassing both the [C11]OHB and the [C11]CO_2_ input and tissue kinetics would have to be developed.

## Conclusion

In summary, the ketone radiotracer [^11^C]OHB promises expanded imaging insights into ketone metabolism in humans due to its favorable dosimetry, rapid clearance from the circulation, anticipated biodistribution, and well defined tissue kinetics. The effective dose after intravenous administration was 3.28 $\upmu$Sv/MBq and 12.51 $\upmu$Sv/MBq after oral ingestion. Tissue [^11^C]OHB kinetics were best analyzed using reversible compartment models and the input function should be corrected for [^11^C]CO_2_.

## Data Availability

The datasets used and analysed during the current study are available from the corresponding author on reasonable request.

## Abbreviations

[^11^C]OHB: (*R*)-[1-^11^C]β-hydroxybutyrate
PET/CT: Positron emission tomography/computed tomography
OHB: 3-Hydroxybutyrate
TAC: Time-activity curves
D-WB: Dynamic whole-body
VOIs: Volumes of interest
TIACs: Time-integrated activity coefficients
AIC: Akaike information criterion
vB: Tissue blood volume fraction
Vt: Volume of distribution
MCTs: Mono carboxyl transporters
RBCs: Red blood cells
IDIF: Image derived input function
1T: 1-Tissue-compartment model
2T: 2-Tissue-compartment model
